# Common wall lizards learn familiar-unfamiliar identity of conspecifics through chemical cues

**DOI:** 10.3758/s13420-025-00670-7

**Published:** 2025-03-06

**Authors:** Roberto Sacchi, Anita Curti, Paola Tassone, Benedetta Chiello, Stefano Scali, Marco Mangiacotti

**Affiliations:** 1 Dipartimento di Scienze della Terra e dell’Ambiente, Università degli Studi di Pavia, via Taramelli 24, 27100 Pavia, Italy; 2 Museo di Storia Naturale di Milano, Corso Venezia, 55, 20121 Milano, Italy

**Keywords:** Individual recognition, Chemical cue, Signal structure, Learning

## Abstract

Despite numerous studies on individual recognition having been carried out on lizards, a clear demonstration that lizards are able to identify conspecifics is still lacking. Individual recognition in lizards involves identifying conspecifics based on distinctive characteristics, including physical, acoustic, and chemical cues. Lizards use specialized epidermal glands for intraspecific communication, which secrete a mixture of proteins and lipids. To demonstrate individual recognition, a training period needs to be devised to establish associations between traits and memories of interactions with other individuals. We thus performed a 3-week study on the common wall lizards (*Podarcis muralis*) to assess whether lizards are able to associate between previous experience with conspecifics and their chemical signals. Further, we investigated whether proteins played a role in this association. We acclimated 40 males to laboratory conditions during the first week. In the second week, we trained lizards to develop familiarity with odors (feces, urine, skin, femoral gland secretion) from previously unknown individuals. During the third week, we tested lizards by exposing them to odors from familiar and unfamiliar individuals. Lizards examined unfamiliar signals for longer in terms of time and frequency compared to familiar ones. These results form the basis of showing that lizards may be capable of recognizing conspecifics as different individuals, based on their chemical signals, even if the observed discrimination remains at the level of familiarity and unfamiliarity. The experiment does, however, demonstrate evidence of learned responses in common wall lizards.

**Supplementary Information:**

The online version contains supplementary material available at 10.3758/s13420-025-00670-7.

## Introduction

Individual recognition (IR) is defined as the ability to identify a conspecific based on distinctive characteristics, including physical features, as well as acoustic and chemical cues (Bradbury & Veherencamp, 2011; Dale et al., 2001). Lizards exhibit complex social systems in which the ability to recognize individuals strongly impacts reproductive success (Fox et al., 2003). For example, individual recognition forms the basis for territorial behavior and dominance hierarchies, where the capacity to convey identity-related information to conspecifics provides an advantage in fine-tuning intraspecific behaviors, and fostering decision-making processes (Bradbury & Veherencamp, 2011; Dale et al., 2001; Johnstone, 1997). Inbreeding avoidance (Berger, 1997), offspring recognition (Stoffel et al., 2015), sexual display modulation (Baeckens et al., 2016), and aggressiveness adjustment (Ancillotto & Russo, 2014) are just a few additional examples of biologically relevant contexts where IR may play a pivotal role. Lizards extensively employ the chemical modality for intraspecific communication (Baeckens, 2019; Martín & López, 2010). This behavior arises from a combination of fine sensory structures, such as the vomeronasal organ coupled with tongue-flicking behavior (Schwenk, 1995), and specialized epidermal glands located in the precloacal region or along the inner part of the thighs (Cole, 1966). The waxy gland secretions play a crucial role in IR. They allow lizards to discriminate their own scent from that of other individuals (Aguilar et al., 2009; Alberts, 1992b; Baird et al., 2015; Mangiacotti et al., 2020, Mangiacotti et al., 2019b), and to discriminate familiar versus unfamiliar conspecifics (Alberts & Werner, 1993; Aragón et al., 2001; Font & Desfilis, 2002; López et al., 2002).

Despite much work being done on recognition processes on lizards (though significantly fewer than in birds and mammals), the majority of studies demonstrated that lizards are capable of discerning among signals from different kind of individuals (such as familiar vs. unfamiliar or neighbor vs. stranger), but this ability does not necessarily imply full IR (Johnston & Bullock, 2001). Merely distinguishing signals from different individuals does not guarantee the ability to identify them as unique entities (Barrows et al., 1975; Falls, 1992; Johnston & Bullock, 2001). IR ultimately develops through the association between specific information (cues) acquired during repeated interactions and the memories of those interactions (Johnston & Bullock, 2001). This distinction lies between the ability to discriminate between familiar and unfamiliar groups of individuals and the concept of recognizing individuals as distinct entities (Cheney & Seyfarth, 1982; Zuberbühler et al., 1999).

The approaches used thus far to demonstrate IR in lizards (e.g., through neighbor-stranger or habituation/dishabituation designs) have primarily focused on behavioral rather than cognitive levels. These methods measure the behavioral response to conspecific signals without considering the prior experiences of focal individuals with those signals. As a result, they have not fully addressed the question of recognizing conspecifics as unique entities. A fundamental step in assessing whether lizards are capable of recognizing conspecifics is to ascertain if they form associations between their previous experiences with conspecifics and their signals or traits, notably, chemical secretions. To do so, it is necessary to devise a training period aimed to establish associations between multiple traits with memory of the interactions with other individuals, before testing signal discrimination (including through neighbor-stranger or habituation/dishabituation designs).

The encoding of identity into chemical signals by lizards is a second relevant topic related to IR. Signal design theory predicts that identity signals should maximize the between-individual variation and minimize the within-individual variation to function properly (Beecher, 1982, 1989). Experimental evidence confirms that traits involved in IR are subject to negative-frequency selection, promoting increased phenotypic diversity and multimodality, and rare traits are favored over more common ones in this context (Tibbetts et al., 2017).

Lizards’ chemical signal, notably the secretions of femoral pores, consist of a mixture of proteins and lipids (Alberts, 1990; Baeckens et al., 2015; Cole, 1966; Mangiacotti et al., 2017), with different proportions according to species and season (Alberts et al., 1992, 2000; Mangiacotti et al., 2019c). Lipids change based on individual condition and likely convey information about the quality of the signaler (Martín & López, 2015), while proteins appear to be the primary carriers of information related to the signaler’s identity (Mangiacotti et al., 2017, 2020, Mangiacotti et al., 2019b).

Lizards serve as an ideal group of model species for testing hypotheses related to IR and how identity is encoded into chemical signals. Firstly, extensive studies have been conducted on individual identity in relation to territorial and social behavior (Aragón et al., 2001; Carazo et al., 2008; Labra & Niemeyer, 1999; Martín & López, 2015). Secondly, given that sexual selection and social interactions play a significant role in controlling territoriality and breeding output (Baeckens & Whiting, 2021; Whiting & While, 2017), the need for an unbiased communication system is reinforced (Alberts, 1992a; Bradbury & Veherencamp, 2011). For instance, green iguanas (*Iguana iguana*) are capable of discriminating self-, familiar- and unfamiliar secretions, including proteins versus lipids (Alberts & Werner, 1993). Similarly, the common wall lizards (*Podarcis muralis*) utilize the protein fraction of femoral secretions to distinguish between self and non-self cues (Mangiacotti et al., 2019b). Recently, we found that male Iberian rock lizards (*Iberolacerta cyreni*) can discriminate proteins from femoral gland secretions and differentiate their own proteins from those of conspecific males (Mangiacotti et al., 2020). However, despite the ability to detect their own proteins amongst others not present in femoral secretions (e.g., bovine serum globulin; Mangiacotti et al., 2020), Iberian rock lizards were unable to distinguish different unfamiliar males. This lack of discrimination may be due to a lack of prior familiarity with the donors before they could be recognized as individuals (i.e., we still relied on a behavioral rather than a cognitive approach).

The present experiment aimed to test whether male common wall lizards can recognize conspecifics based on their chemical signals and whether proteins play a role in this process. We employed a cognitive approach by training individuals to become familiar with the chemical signals of previously unknown conspecifics. This approach allows us to establish whether lizards possess a key prerequisite for hypothesizing that they are capable of recognizing conspecifics as unique entities.

## Material and methods

### Lizard collection, handling, and housing

Forty adult male common wall lizards (SVL range: 54–74 mm) were captured during spring 2022 (22 March–17 April) in South-Western Lombardy, Northern Italy: 34 were captured in and around the town of Pavia (45°11′N, 9°9′E), two were from the town of Voghera (44°59′N, 9°0′E), and four were from the town of Lentate sul Seveso (45°40′N, 9°7′E). Within 2 h of capture, lizards were transferred to the University lab, measured for their snout-to-vent length (SVL; to the nearest mm), and weighed (± 0.1 g). Then, the secretions from the femoral glands were collected by applying a gentle pressure along the thighs, with the help of a steel spatula, until all the glands (both legs) were emptied. Secretions were stored into glass vials at −20 °C until chemical analyses (Mangiacotti et al., 2017). We individually housed lizards in 20 × 30 × 20 cm transparent plastic enclosures (Sacchi et al., 2015) with a plastic tube fixed to a tile with cable ties as shelter/basking site, a small clay dish for food, and a small bowl of water (50 cc). Enclosures were kept at a temperature of 28 °C with 45-W heating mats (Vivarium Heat Mat, 80 × 28 cm, 220–240 V, Herpstore), and illuminated with white LED lamps. Mats and lights were turned on at 8.00 and turned off at 18.00 thanks to a timer (TFA, Thermo-Timer). The housing room was maintained around 21 °C. We did not supply any additional source of UV since the lizards were housed for only 3 weeks, and then released. However, the housing room was equipped with windows that were not opaque to the UV. A heat source was placed in one half of the plastic enclosure so that each individual had a warm spot for basking (around 30 °C) and an unheated area (around 21 °C) to cool down. We did not control for humidity gradient, but we refilled the water bowl every day. We released all lizards at their capture sites following use in trials, no more than 3 weeks after their capture date. All enclosures were carefully cleaned (with alcohol and paper towels) before a new individual was placed into it to remove any chemical cues of the previous subject. No lizard was injured or killed during the study, and all lizards looked healthy at release.

### Protein extraction

The soluble proteinaceous fraction of the femoral pore secretions was extracted following the same procedure used in previous studies (Mangiacotti et al., 2017), i.e., first adding 200 μl of n-hexane to the waxy secretion to complete defatting, vortexing for 2 min, and then centrifuging at 6,000 *g* for other 2 min (Mangiacotti et al., 2019b). The supernatant was removed, and the pellet airdried. This procedure was repeated three times. Afterwards, 1 ml of 10 mM (pH 7.4) phosphate-buffered saline (PBS) was added to the dry pellet. After vortexing and centrifuging, the supernatant containing the soluble proteins was recovered and stored in a freezer (−20 °C) until be used in the experimental trials.

### Experimental procedures

Each lizard was kept in the laboratory for three consecutive weeks. The first week was spent acclimating to the laboratory conditions. On the second week, we familiarized males with the odors (feces, urine, skin, femoral gland secretion) of a previously unknown lizard, i.e., not belonging to the same location (see detail below). On the third week, we tested the lizards by exposing them to chemical signals from the familiar males and from a second unfamiliar individual of the same location of the familiar one.

**Fig. 1:**
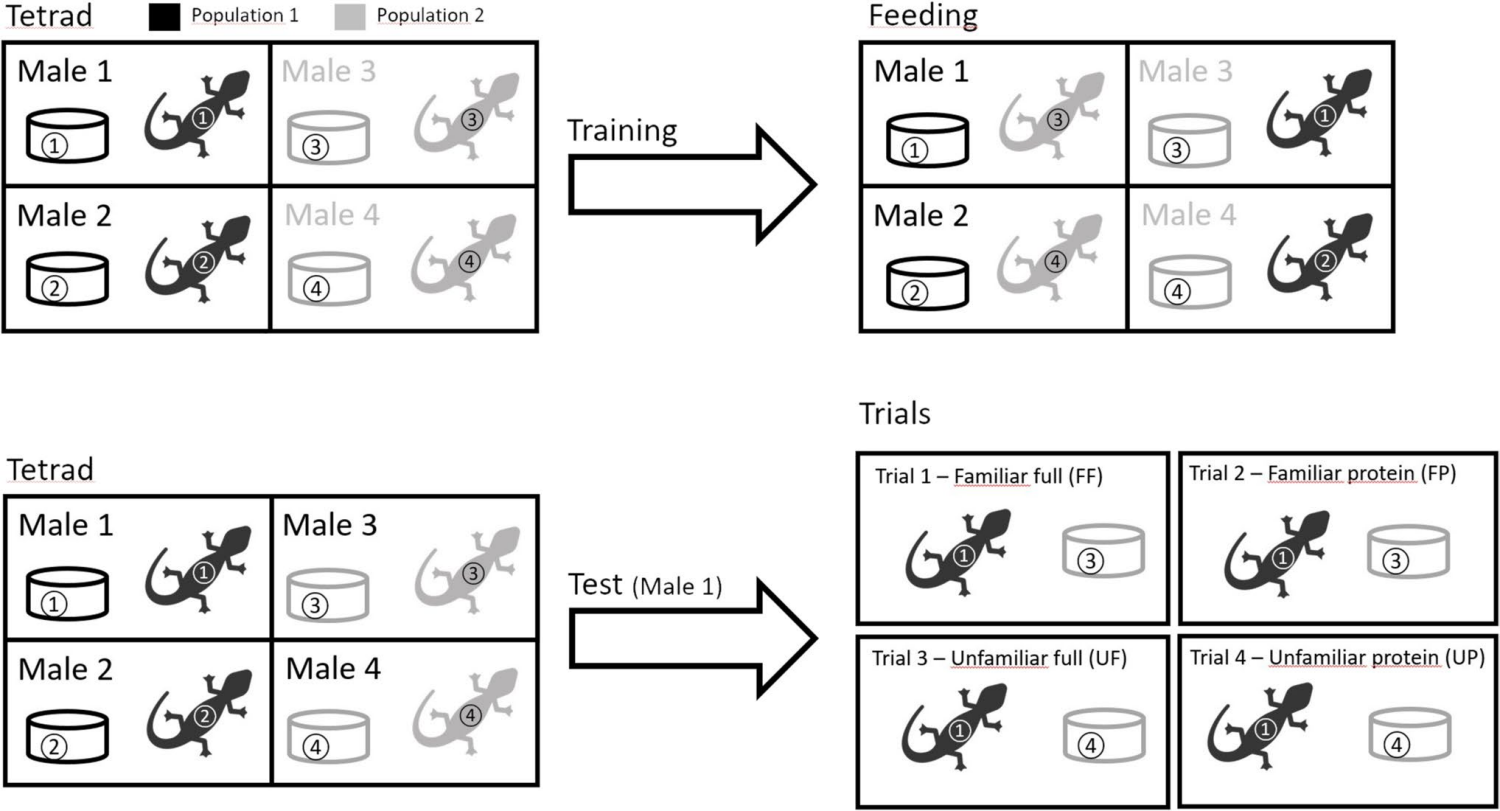
Diagram of the experimental setup used to create familiarity with an unknown conspecific (training), and test for discrimination between the complete set of chemical signals (feces, urine, skin, femoral gland secretion) of the familiar individual of the tetrad (“familiar full”; FF), the complete signal of the unfamiliar individual of the tetrad (“unfamiliar full”; UF), the protein fraction of the femoral pore secretion of the familiar individual of the tetrad (“familiar protein”; FP), and the protein fraction of the femoral pore secretion of the unfamiliar individual of the tetrad ("unfamiliar protein”; UP)

During the acclimation week (run-in), just after capture, lizards were assigned to groups of four individuals (“tetrads” hereafter; Fig. 1), by randomly choosing two pairs of individuals, each from a different sampling site. Thus, for each individual in a tetrad, the two individuals of the other pair were identified as the familiar and the unfamiliar one, and each individual in a tetrad was used as familiar or unfamiliar from the males of the opposite pair. Lizards were collected on Monday of the acclimatation week, every 2 days starting from the capture day (i.e., on Monday, Wednesday, and Friday), but not on Sunday. Fasting serves to make lizards more prone to exploring the cage of the familiar male during the training on the next Monday.

During the training week (from Monday to Sunday), lizards were trained to become familiar with one of the two individuals in the other pair of the same tetrads (training). Familiarity was achieved by feeding the focal male only within the enclosure of the familiar male (Fig. 1), for each day of the training period. Before the light and heating mats turned on, around 8.00 a. m., all males were closed inside the shelter (where lizards were usually still present) with a foam plug and moved into the familiar enclosure (this ensured that all lizards were in contact with odors of the familiar individual without being in physical contact with him). After the lamps and mats were switched on, a mealworm was placed in the clay dish, and the foam plug removed. Each training session lasted an hour, and we regularly checked whether the focal male had left the shelter and fed. The first check was done after 5 min following the plug removal, and the subsequent ones at 15-min intervals. If the lizard did not leave the shelter for the duration of the training and/or it did not feed, the feeding time was arbitrarily set to 100 min (i.e., a long time). Once the training ended, the focal individuals were returned to their own enclosure. As for the run-in, lizards were still fasting on Saturday and Sunday to make them more prone to explore the arena during the tests on the following Monday.

During the test week (from Monday to Thursday), lizards were tested in a new arena (60 × 40 cm) to measure the behavioral response to four different chemical stimulations (Fig. 1): the complete set of chemical signals (feces, urine, skin, femoral gland secretion) of the familiar individual of the tetrad (“familiar full”; FF), the complete signal of the unfamiliar individual of the tetrad (“unfamiliar full”; UF), the protein fraction of the femoral pore secretion of the familiar individual of the tetrad (“familiar protein”; FP), and the protein fraction of the femoral pore secretion of the unfamiliar individual of the tetrad ("unfamiliar protein”; UP). The experiments were performed in two identical arenas (60 × 40 cm) into which the lizards were moved from their own enclosure only 10 min before the trial. Lizards were moved to the arena with the same procedure used for training, and the shelter tube was inserted into a slot on the short side of the arena, so that when removing the cap the lizard could freely enter the arena. Before starting the trial, we put on the opposite side of the entrance of the focal lizard a clay dish identical to that used for feeding containing the chemical stimulation. We used the dish inside familiar and unfamiliar enclosures for FF and UF, while we homogeneously dispersed 250 µl of protein solution on new and cleaned clay dish for FP and UP. Further, a mirror was used to simulate the arrival of a rival male within the arena, in order to trigger the behavioral response of the focal males. We had previously shown that common wall lizards perceive their own mirror image as a rival, and behave aggressively in response, sometimes even biting (Sacchi et al., 2021; Scali et al., 2019). To avoid visual disturbance during the trials, the plastic sides (20 cm in high) of the two arenas were uniformly opaque. Before each trial, the male was heated for 2 min using a 75 W halogen infra-red lamp (Reptiles-Planet.com) positioned 40 cm above the arena. The movements of the lizard were recorded using two webcams (ELP-USB8MP02G-SFV, 5–50 mm, with 8 megapixels resolution), one for each arena, mounted 80 cm above the arena, and connected to two PCs by a 3-m cable. The arenas were illuminated by two white LED lamps (OSRAM Biolux L36-W/965). Recording was managed by OBS Studio software v29.0.21.0.0.1, setting quality to 1,280 × 1,024 pixels and 15 frames per second (fps). Recording duration was set to 15 min (27,000 frames) after the focal male had touched the dish containing the chemical stimulus. At the end of the trial the body temperature was measured with a handheld infra-red thermometer (Lafayette TRP-39, sensitivity: 0.1 °C; precision: ± 2%), and the lizard was moved into its own enclosure. Each individual performed only one experiment per day (thus, we carried out a total of eight trials per day), between 11:00 a.m. and 2:00 p.m. The order of the chemical stimuli, as well as the arena used for the trial, were balanced so that each lizard did two trials per arena, and so that the four chemical stimuli were equally distributed on the experimental sequence. We repeated a trial the subsequent day if the lizard did not move after 10 min from the start. Overall, we performed 160 trials between 3 April and 5 May 2023.

When on a regular basis, we kept 24 lizards in the laboratory at a time: two tetrads followed acclimatization, two tetrads were undergoing training, and two tetrads were under testing. The experiment ended after completing five cycles of two tetrads each, involving a total of 40 lizards. Nevertheless, one individual never ate during the training and was then excluded, leading to a final sample of 39 lizards used in the following analyses.

### Response variables

We used DORIS, Detection of Object and Tracking (Friard & Gamba, 2016), to analyze video files and extract the tracks of the focal lizards into the arenas. From tracks we assessed the duration (in seconds) and frequency (number every minute) of contacts by the focal lizards with the clay dish with the chemical stimulus.

### Statistical analyses

To assess if the lizards got used to the chemical signals of the familiar individual during the training we used a random intercept linear mixed model (LMM). The time spent eating the mealworm was the dependent variable and the training day (first to sixth, code as six-level factor) and body size (SVL) were the fixed effects. The tetrad, the individual (nested within tetrad), and the identity of the donor of the chemical stimulus entered the model as random effects.

Then, we used the same model to examine if lizards responded differently, one for each response variable (i.e., duration and frequency of contacts with the stimulus). Fixed effects were the treatment (familiar vs. unfamiliar), the stimulus (full vs. protein), and their interaction. We also added as fixed effects the arena (A vs. B), the body size (SVL), the body temperature, and the trial (first to fourth) as controlling effects accounting for the experimental setting, lizard age, and the sequence of stimulation. Continuous variables were standardized by subtracting the mean and dividing by the standard deviation. The tetrad, the individual (nested within tetrad), and the identity of the donor of the chemical stimulus entered the model as random effects. LMMs were fit in a Bayesian analytical framework using STAN as available in the package brms (Bürkner, 2017), using flat priors for coefficients, intercepts, and standard deviations (μ = 0 and σ = 100). For all models, Markov chain Monte Carlo parameters were set as follows: number of independent chains = three; number of iterations = 34,000; burning = 4,000; thinning = three. We checked convergence through trace plot and autocorrelation along chains, and results from the posterior distribution are reported as the half sample mode (HSM, Bickel & Frühwirth, 2006) with 95% and 50% highest density intervals (HDI_95_ and HDI_50_; Kruschke, 2010). All analyses were done in R 4.2.1 (R Core Team, 2022) using the package HDInterval (Meredith & Kruschke, 2018).

## Results

### Training

**Fig. 2:**
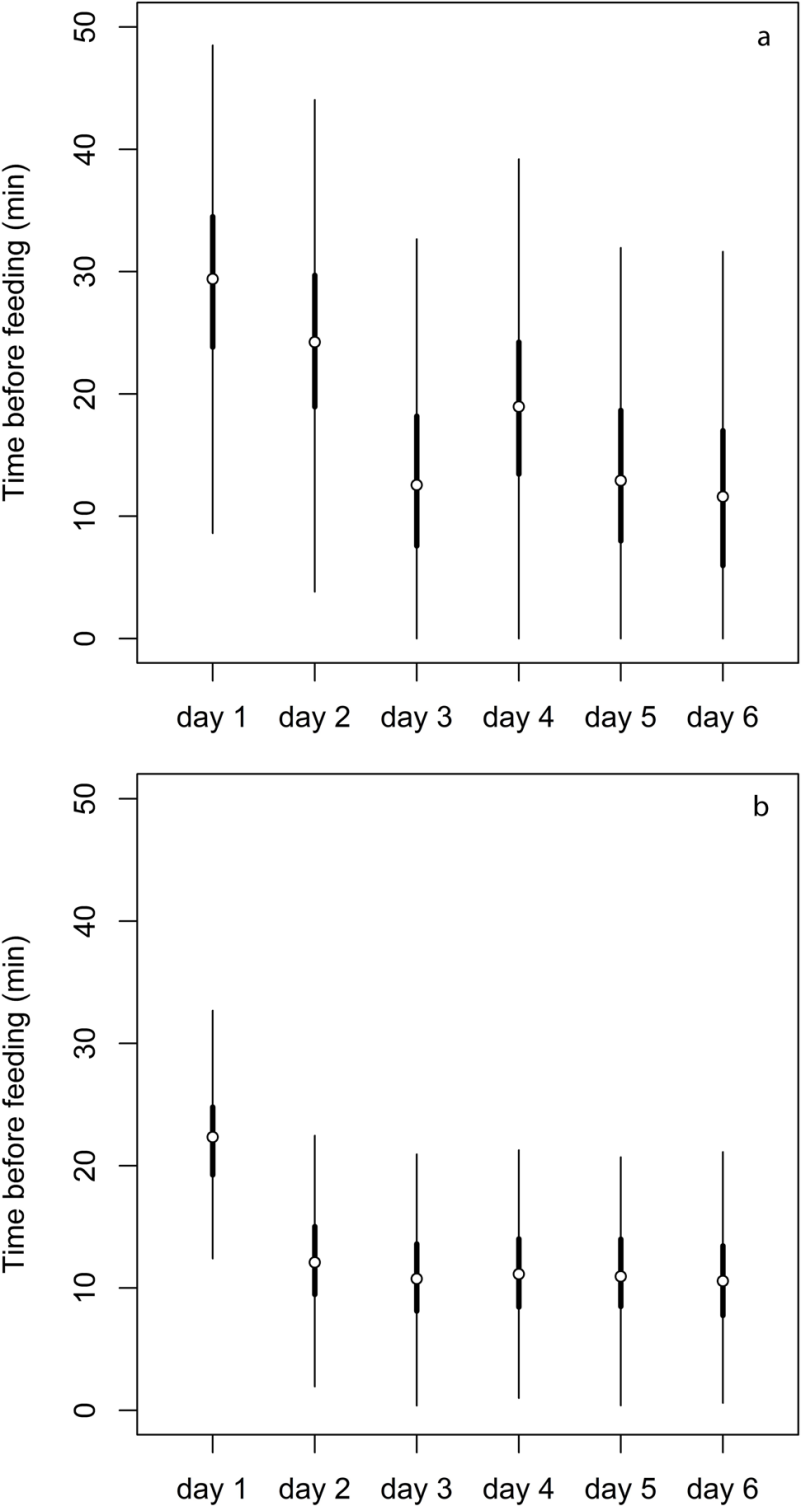
Bayesian model predictions for the time spent eating the prey (a mealworm) by common wall lizard males during the hour of training to create familiarity with the chemical signal of donors (see *Methods* for details). Predictions in the left panel include values fixed at 100 s for liz ards that did not eat the prey, which were excluded for the predictions in the right panel. Circles indicate half sample mode (HSM), and thick and thin lines represent HDI_50_ and HDI_95_(50% and 95% highest density intervals), respectively

In the first training day, lizards took an average of 30 ± 5 min to catch the prey, and four individuals did not leave the shelter. On the second day, the feeding time decreased to 24 ± 5 min, with six lizards still not leaving the shelter. Starting from the third day, the time for catching mealworm fell below 20 min (ranging from 13 ± 2 to 19 ± 4 min), and one to four lizards continued to remain in the shelter. The LMM (Fig. 2a) confirmed that the time spent before eating decreased from the first to the second day (β = −5.1 ± 4.2; HDI_95_ = −12.2, 1.9; P_β<0_ = 0.88), followed by a further decrease on the third day (β = −11.7 ± 4.2; HDI_95_ = −18.7,−4.8; P_β<0_ > 0.99). Subsequently, the time slightly increased from the third to the fourth day (β = 6.4 ± 4.2; HDI_95_ = −0.5,13.3; P_β<0_ = 0.06), then decreased again on the fifth day (β = −6.0 ± 4.2; HDI_95_ = −12.9, 0.9; P_β<0_ = 0.92), and finally remained unchanged on the last day (β = −1.4 ± 4.5; HDI_95_ = −8.9, 6.1; P_β<0_ = 0.62).

To check the potential effects of adjusting the time for focal lizards that did not leave the shelter at 100 min, we repeated the analysis after excluding those data. The second LMM yielded very similar results (Fig. 2b): feeding time decreased from 22 ± 3 min to 11 ± 2 min between the first two days of training (β = −10.2 ± 2.5; HDI_95_ = −14.3,−6.2; P_β<0_ > 0.99), remaining consistently stable at 10 ± 2 to 12 ± 2 min in the subsequent days (β < 1.3 ± 2.4; P_β<0_ < 0.71).

### Test

**Fig. 3:**
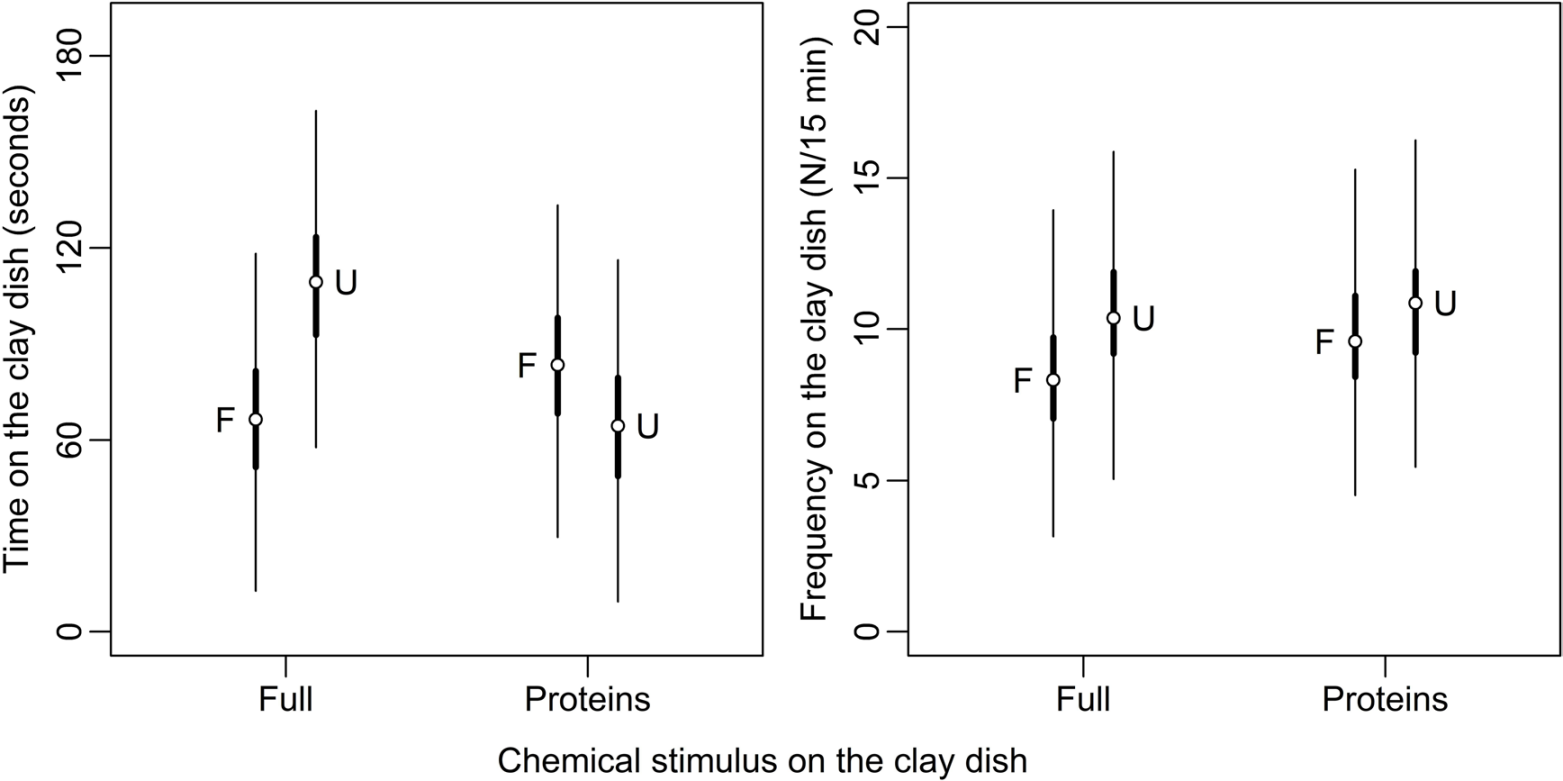
Bayesian model predictions for the responses of common wall lizard males to full (lipids + proteins) and the protein fraction femoral pore secretion of familiar (F) and unfamiliar (U) male donors (see *Methods* for details on the training to obtain familiarity). Circles indicate half sample mode (HSM), and thick and thin lines represent HDI_50_ and HDI_95_ (50% and 95% highest density intervals), respectively

In all trials, males made contact with the clay dish (frequency range: 1–49) and spent on average 77 ± 94 s (range: 2–478 s) interacting with it. The conditional effects of the LMM for the time spent by focal lizards on the clay dish (Fig. 3) revealed that individuals allocated 43 ± 16 s (HDI_95_ = 12,73; P_β>0_ > 0.99) more time to the unfamiliar stimulus than to the familiar one when faced with the full signal (UF vs. FF; Fig. 3). In contrast, a different pattern emerged with the protein fraction of the signal: males spent 18 ± 16 s (HDI_95_ = −49,13; P_β<0_ = 0.89) less time with the unfamiliar signal compared to the familiar one. However, the protein fraction was equally effective as the full signal in attracting focal males (Fig. 3). Notably, males dedicated more time exploring the FP than the FF signal (17 ± 15 s; HDI_95_ = −13,47; P_β>0_ = 0.89), and no appreciable differences were evident when comparing the males’ responses to FF and UP (2 ± 15 s; HDI_95_ = −32,30; P_β>0_ = 0.45). While limited effects were detected for the control variables (SVL, area, and trial) included in the fixed component of the model (Online Supplemental Material (OSM) Table S1), the random component of the LMM (OSM Table S2) showed no evident effects except for the individuals, which exhibited high repeatability (Intraclass Correlation Coefficient: 0.49 ± 0.10, HDI_95_ = 0.31,0.64).

The same analysis on the frequency of contact with the clay dish confirmed the results obtained with the previous model for the full signal, but it yielded different results for the stimulation with proteins (Fig. 3). Focal males explored the dish more frequently (2.0 ± 1.2 times/15 min; HDI_95_ = 0.1,4.0; P_β>0_ > 0.99) when encountering the unfamiliar stimulus compared to the familiar one in the context of the full signal. Similarly, with the protein stimulus, males visited the clay dish 1.3 ± 1.2 times/15 min more with the unfamiliar signal than the familiar signal (HDI_95_ = −0.7,3.2; P_β>0_ = 0.86). As in the previous analysis, the protein fraction was equally effective as the full signal in attracting focal males (Fig. 3). Specifically, males explored the clay dish approximately 1.3 ± 1.2 times/15 min more with the FP signal than with the FF signal (HDI_95_ = −1.1,3.5; P_β>0_ = 0.85), and 2.5 ± 1.2 times/15 min more with the UP signal than with the UF signal (HDI_95_ = 0.6–4.4; P_β>0_ = 0.98). Consistent with the previous model, only limited effects were detected for body size (SVL), area, and trial (OSM Table S1). Finally, the random component of the model (OSM Table S2) revealed high repeatability for both individual lizards (ICC: 0.49 ± 0.09, HDI_95_ = 0.28,0.59) and tetrads (ICC: 0.36 ± 0.26, HDI_95_ = 0.03,0.88).

## Discussion

Through a cognitive approach, we clearly demonstrated that common wall lizards can form an association between a high variable signal – the secretion of femoral pores – and a positive experience, such as feeding on a mealworm. This association makes lizards able to distinguish between familiar and unfamiliar individuals only using information encoded into chemical signals. Specifically, when interacting with conspecifics’ complete chemical signals (including urine, feces, femoral pore secretions and other chemicals produced by the lizard’s body), lizards examine the signals from unfamiliar individuals for longer, both in terms of time and frequency of contact, compared to the familiar ones. These results make the bases for arguing that lizards are actually capable of recognizing two conspecifics as unique entities. Nevertheless, our experiment does not completely rule out the possibility that discrimination between the two chemical alternatives we offered in the tests (i.e., FF vs. FU and PF vs. PU) was simply a discrimination between contexts rather than a recognition between individuals. To clarify this possibility, we should analyze the discriminatory abilities of lizards after training them with novel, non-social chemicals. However, previous experiments using novel chemicals have shown that femoral pore secretions mediate responses linked to the identity of lizards, allowing, for example, self- as opposed to non-self-discrimination (Mangiacotti et al., 2020, Mangiacotti et al., 2019b). Further support for the hypothesis that femoral pore secretions mediate identity is offered by comparing the responses of lizards have when faced with secretions and their mirrored images (Scali et al., 2019). Indeed, lizards are less frightened by self-chemicals (i.e., they move), but are unable to recognize themselves in mirrored images, and attack them as a rival (Coladonato et al., 2020; Sacchi et al., 2021; Scali et al., 2019). Therefore, our data provide a strong indication that discrimination of chemical signals in behavioral tests might be linked to IR, although the evidence we produced cannot be considered conclusive.

A second relevant point worth noting is that the discrimination we observed in our experiment remains at the level of familiarity and unfamiliarity. During training, the lizards were exposed to only one individual, leading to a choice between an individual known through the association with food and one never perceived. Thus, the discrimination demonstrated is still at the group rather than the individual level. Nevertheless, the experiment shows that lizards can associate an experience (such as reinforcement with food) with a chemical signal, influencing their choices. This is a cognitive response because the male donors were entirely unknown to the focal males, functioning both as a familiar and as unfamiliar entities based exclusively on the experimental protocol. Consequently, we can be highly confident that the observed responses depend on the association between the reinforcement and individual signal features rather than the features of the signal itself.

These results highlight the relevance of the experimental design, particularly the cognitive approach, in detecting individuality in the behavioral responses of animals. In our prior experiment involving *Iberolacerta cyreni*, we failed to detect IR when the scent of two unfamiliar males in a habituation-dishabituation experimental setup elicited the same level of interest (i.e., tongue flicking), with no discernible signs of discrimination (Mangiacotti et al., 2020). Although the chemical signal (specifically the proteinaceous fraction) did not suffice to trigger IR, we cannot rule out the possibility that lack of recognition resulted from insufficient information acquired during prior interactions with the unfamiliar individuals. Our current experiment supports this second conclusion by demonstrating that discrimination emerges only after a training phase, during which associations form and allow individuals to effectively discriminate. From this perspective, the habituation-dishabituation protocol is ill-suited for investigating the ability to differentiate between stimuli whose effects may depend on an individual’s prior experience with those stimuli, as is the case with IR.

From the perspective of the signal structure, we clearly found that the proteinaceous fraction alone is able to trigger a similar response to the full signals, and consequently might convey IR-related information by itself. This result is relevant in the perspective of the comprehension of identity signals in animals, notably lizards. Indeed, identity signals of whatever structure – visual, olfactory, or acoustic – share the same properties, i.e., a set of hyper-variable characteristics that collectively provide the receiver with a unique signature of the sender (Tibbetts & Dale, 2007). The variability of proteins occurring in femoral secretions is extremely wide, and is potentially useful for discriminating species and population, from groups of individuals up to individuals within a population (Mangiacotti et al., 2017, 2021, Mangiacotti et al., 2019a, Mangiacotti et al., 2019b). Therefore, proteins from femoral secretions can be used to form cognitive associations between memories of past experiences and the unique protein assemblage in each individual, leading to identity signals. Associations between experience and odors form the base for IR in golden hamsters, *Mesocricetus auratus* (Johnston & Bullock, 2001). In this species, individuals have many different distinctive odors that naïve receivers treat as if they are from different individuals. Experience with the live scent donor enables hamsters to treat the odors as if they represent different individuals, suggesting that the association between odors and experience leads to the formation of cognitive representations of other individuals (Johnston & Bullock, 2001). In this sense, the vomeronasal organ in vertebrates primarily evolved for the detection of pheromones, but adapted in order to also detect non-pheromonal stimuli, including molecules responsible for smell (Silva & Antunes, 2017). Detection in this organ is based on two main classes of receptors, the vomeronasal receptor (VR) type-1 (V1R) and type-2 (V2R), the former devoted primarily to volatile molecules, the latter to water-soluble oligopeptides (Silva & Antunes, 2017). V2Rs are extremely sensitive receptors that can respond to small oligopeptides at concentrations as low as to 10^−13^ M (Leinders-zufall et al., 2000). Interestingly, V2Rs are the dominant receptors in the vomeronasal organs of reptiles (Silva & Antunes, 2017). Considering that vomeronasal detection is strongly associated with tongue flick, there are reason to assume that the proteinaceous component of the femoral secretions, notably the oligopeptides, may form the structural component on which the IR is based.

In conclusion, our results show how lizards are capable of displaying sophisticated cognitive abilities, which are comparable to those achieved by birds and mammals. More generally, with this study we contribute to the “rehabilitation” process of reptile reputation (Font et al., 2023), traditionally seen as behaviorally and cognitively undeveloped. In fact, there is increasing evidence that reptiles have rich, complex behavioral repertoires based on cognitive functions, rather than simple stereotyped modules (Font et al., 2023). Our work provides a further step in this new perspective.

## Supplementary Information

Below is the link to the electronic supplementary material.
**ESM 1** (DOCX 14.5 KB)

## Data Availability

The authors will make accessible the original dataset of the analyses on a public repository before the final publication.
