# Supplementary material for: Common wall lizards learn familiar-unfamiliar identity of conspecifics through chemical cues
Source: Learn Behav. 2025 Mar 6;53(3):265–74. doi: 10.3758/s13420-025-00670-7 (PMC12408672; doi:10.3758/s13420-025-00670-7)
Supplement: Supplementary file 1 — Below is the link to the electronic supplementary material. ESM 1 (DOCX 14.5 KB) [file 13420_2025_670_MOESM1_ESM.docx]

**Table S1.** Coefficients of the Bayesian models used to analyse the response of lizards to the familiar/unfamiliar treatment for full and proteinaceous signals.

| **Fixed component** | **β ± SE** | **HDI_95_** | **P_β>0_** |
| --- | --- | --- | --- |
| **Duration** |  |  |  |
| Intercept | 51.2 ± 29.9 | -7.9, 109.1 | 0.964 |
| Treatment (Unfamiliar) | 42.8 ±15.6 | 12.1, 73.2 | 0.997 |
| Stimulus (Protein) | 17.0 ± 15.4 | -13.5, 46.7 | 0.867 |
| Trial | 6.5 ± 5.0 | -3.4, 16.1 | 0.903 |
| SVL | -12.7 ± 12.5 | -37.2, 12.3 | 0.151 |
| Arena (B) | -4.3 ± 11.1 | -26.1, 17.4 | 0.345 |
| Treatment × Stimulus | -61.7 ± 22.0 | -105.8, -19.2 | 0.003 |
| **Frequency** |  |  |  |
| Intercept | 6.1 ± 2.8 | 0.7, 11.8 | 0.973 |
| Treatment (Unfamiliar) | 2.0 ± 1.2 | -0.3, 4.3 | 0.958 |
| Stimulus (Protein) | 1.2 ± 1.2 | -1.1, 3.5 | 0.854 |
| Trial | 0.9 ± 0.4 | 0.1, 1.6 | 0.988 |
| SVL | 0.3 ± 0.9 | -1.4, 2.1 | 0.652 |
| Arena (B) | 0.4 ± 0.8 | -1.3, 2.1 | 0.696 |
| Treatment × Stimulus | -0.7 ± 1.7 | -4.0, 2.5 | 0.327 |

**Table S2.** Random effects of the Bayesian models used to analyse the response of lizards to the familiar/unfamiliar treatment for full and proteinaceous signals.

| **Random component** | **σ** | **ICC** |
| --- | --- | --- |
| **Duration** |  |  |
| Tetrad | 30 (3 - 99) | 0.18 (0.003 – 0.73) |
| Tetrad/individual | 64  (46 – 86) | 0.49  (0.31 – 0.65) |
| Stimulus | 15 (1 – 41) | 0.05 (0 – 0.28) |
| Error | 66  (59-74) |  |
| **Frequency** |  |  |
| Tetrad | 3.2  (0.8 – 11.6) | 0.36 (0.02 – 0.88) |
| Tetrad/individual | 4.4  (3.3 – 5.8) | 0.49 (0.28 – 0.59) |
| Stimulus | 0.6  (0.1 – 1.7) | 0.04 (0 – 0.10) |
| Error | 5.0  (4.5 – 5.6) |  |
